# Efficacy of T2 Magnetic Resonance Assay in Monitoring Candidemia after Initiation of Antifungal Therapy: the Serial Therapeutic and Antifungal Monitoring Protocol (STAMP) Trial

**DOI:** 10.1128/JCM.01756-17

**Published:** 2018-03-26

**Authors:** Eleftherios Mylonakis, Ioannis M. Zacharioudakis, Cornelius J. Clancy, M. Hong Nguyen, Peter G. Pappas

**Affiliations:** a Infectious Diseases Division, Warren Alpert Medical School of Brown University, Providence, Rhode Island, USA; b Department of Medicine, Warren Alpert Medical School of Brown University, Providence, Rhode Island, USA; c University of Pittsburgh Medical Center, Pittsburgh, Pennsylvania, USA; d University of Alabama at Birmingham, Birmingham, Alabama, USA

**Keywords:** candidemia, invasive candidiasis, molecular diagnostics, monitor, T2 Candida, T2MR

## Abstract

The performance of blood culture for monitoring candidemia clearance is hampered by its low sensitivity, especially during antifungal therapy. The T2 magnetic resonance (T2MR) assay combines magnetic resonance with nanotechnology to identify whole Candida species cells. A multicenter clinical trial studied the performance of T2MR in monitoring candidemia clearance compared to blood culture. Adults with a blood culture positive for yeast were enrolled and had blood cultures and T2MR testing performed on prespecified days. Thirty-one patients completed the trial. Thirteen of the 31 patients (41.9%) had at least one positive surveillance T2MR and/or blood culture result. All positive blood cultures (7/7 [100%]) had an accompanying positive T2MR result with concordance in the identified Candida sp., while only 7/23 (30.4%) T2MR results had an accompanying positive blood culture. There was one case of discordance in species identification between T2MR and the preenrollment blood culture with evidence to support deep-seated infection by the Candida spp. detected by the T2MR assay. Based on the log rank test, there was a statistically significant improvement in posttreatment surveillance using the T2MR assay compared to blood culture (*P* = 0.004). Limitations of the study include the small sample size and lack of outcome data. In conclusion, the T2MR assay significantly outperformed blood cultures for monitoring the clearance of candidemia in patients receiving antifungal therapy and may be useful in determining adequate source control, timing for deescalation, and optimal duration of treatment. However, further studies are needed to determine the viability of Candida species cells detected by the T2MR assay and correlate the results with patient outcomes. (This study is registered at ClinicalTrials.gov under registration number NCT02163889.)

## INTRODUCTION

Invasive candidiasis is a health care-associated infection with high morbidity and a mortality rate exceeding 40% (1–3). Optimal antifungal therapy and control of the source of infection constitute the cornerstones for successful treatment (4, 5). As part of optimal antifungal therapy, the most recent Infectious Diseases Society of America (IDSA) clinical practice guidelines support follow-up blood cultures every 24 to 48 h in order to monitor clearance of candidemia, determine the need for further interventions for source control, and guide deescalation and the total duration of therapy (6). However, the low sensitivity, suppression by antifungal therapy, and prolonged time to result (3 to 5 days) deem blood cultures a suboptimal tool to guide the treatment of candidemia.

More specifically, despite the reliance on blood culture as a monitoring standard to guide therapy, blood cultures perform poorly for detecting invasive candidiasis. In studies comparing their performance with postmortem autopsy results of patients proven to have invasive candidiasis, the sensitivity of blood cultures ranged from 21 to 71% (7). Moreover, blood cultures are heavily influenced by the initiation of antifungal therapy. For example, in a study examining the performance of two commonly used blood culture systems in seeded blood culture bottles, the addition of therapeutic levels of antifungal agents halved the detection rate of Candida species (8, 9). Even fungus-specific blood cultures have not been shown to outperform regular blood cultures in clinical trials (10–12); also, they are rarely used in clinical practice and are not included in the recently published guidelines for monitoring of candidemia.

The T2 Magnetic Resonance (T2MR) technology platform combines magnetic resonance with nanotechnology to identify whole Candida cells within 3 to 5 h of processing a sample (8, 13). The T2MR assay directly analyzes whole-blood specimens to identify Candida spp. without the need for prior isolation of Candida cells, with a specificity of 99.4% and sensitivity of 91.1% (14). Importantly, early *in vitro* interference studies for exogenous substances have shown that the T2MR assay is not suppressed by the presence of antifungal agents (8, 15). The purpose of this multicenter prospective clinical trial, designated the Serial Therapeutic and Antifungal Monitoring Protocol (STAMP) trial (registered at ClinicalTrials.gov under registration no. NCT02163889), was to investigate the performance of the T2MR assay as a monitoring tool for posttherapy clearance of candidemia compared to blood cultures.

## MATERIALS AND METHODS

### T2 magnetic resonance assay.

The T2MR is a qualitative assay that utilizes the magnetic resonance-based approach used in magnetic resonance imaging (MRI) technology and is run automatically on the T2Dx instrument. Details about this technology can be found in reference 8. In brief, T2Dx lyses Candida cells by mechanical bead beating, uses pan-Candida PCR primers to amplify the internal transcribed spacer 2 (ITS2) region within the Candida ribosomal DNA operon, introduces superparamagnetic nanoparticles coated with binding agents that target species-specific capture probes nested within the pan-Candida amplicons into whole-blood samples, and detects and provides species-level identification by measuring the magnetic resonance signal produced as the result of the agglomeration of the superparamagnetic particles. The T2MR assay has been designed to detect intact Candida cells and not circulating DNA due to the unclear clinical relevance of circulating pathogen DNA as a marker of infection (8).

In order to monitor for the presence of inhibitors, T2MR processes an internal control with each clinical specimen. If the internal control is invalid and there are no positive T2MR signals, an “invalid” result is displayed, indicating the possible existence of an inhibitor that interferes with Candida detection. The T2MR assay is designed to detect 5 Candida spp.: Candida albicans, Candida tropicalis, Candida krusei, Candida glabrata, and Candida parapsilosis. The T2MR results are grouped, and one of 3 results is reported: C. albicans/C. tropicalis, C. krusei/C. glabrata, or C. parapsilosis on the basis of antifungal resistance patterns of the aforementioned Candida species (6).

### Trial design.

The STAMP clinical trial was conducted between September 2014 and April 2017 at 3 centers in the United States. Patients age 18 to 95 years with a blood culture positive for yeast receiving or scheduled to receive antifungal therapy within 12 h from the positive blood culture result were eligible for participation in the study. Patients had to be enrolled within 36 h from the positive blood culture result. Patients receiving any novel drug compound within 30 days prior to potential enrollment were excluded from the study. In cases where the yeast identified in the preenrollment blood culture was different from the 5 Candida spp. targeted by the T2MR technology, patients were withdrawn from the study and no further blood draws were performed. The institutional review board of each center approved the study protocol. Written informed consent was obtained from all patients.

### Sample collection and outcomes.

**FIG 1:**
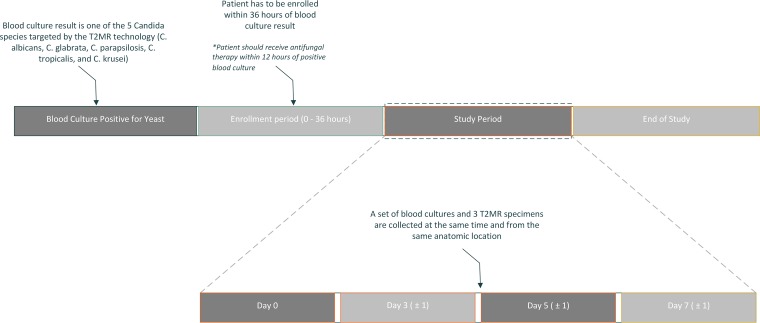
Graphic representation of study design.

A set of aerobic and anaerobic blood cultures and 3 whole-blood T2MR specimens (tubes A, B, and C) were collected from each participant on the day of enrollment (day 0) and then on days 3, 5, and 7 or until hospital discharge (Fig. 1). Only the data of patients who had at least 2 surveillance sets of T2MR specimens and blood cultures collected were used in the study analysis. A window of 1 day was allowed for study visits to account for weekends and holidays. Patients who were discharged before having 2 specimens collected were excluded from the study. The T2MR results were not used for clinical decision-making, and the antifungal regimen was determined in accordance with routine institutional practice.

T2MR specimens and blood cultures were collected at the same time from the same anatomical collection site either through a peripheral venipuncture or from a central line or port. T2MR clinical specimens were collected in K2 EDTA plastic blood collection Vacutainers. Tube A was stored at room temperature (20°C to 25°C) and was analyzed within 12 h of collection. Tube B was refrigerated up to 72 h until tube A was successfully run and was then frozen if not tested. Tube C was maintained in frozen storage (−70°C to −80°C) immediately after collection. The blood cultures were processed in accordance with routine institutional practice for a period of 5 days or until a positive blood culture result was reported, whichever occurred first. The BacT/Alert 3D, Bactec FX, and VersaTREK blood culture systems were used in the participating study centers. In cases of positive blood cultures, the species of the bloodstream isolate was identified with matrix-assisted laser desorption ionization–time of flight mass spectrometry (MALDI-TOF MS), Vitek 2, Microscan, or a peptide nucleic acid (PNA) probe, in accordance with routine institutional practice.

### Statistical analysis.

The collected data were represented as lifetime data, and the Kaplan-Meier estimator was used to measure the length of time patients remained candidemic on the 2 diagnostic methods. A log rank test was used to compare the above-mentioned distributions of the two samples. Categorical data were presented as relative frequencies and were compared using the chi-square test. Statistical significance was set at 0.05. Statistical analysis was performed using the Stata version 14 software package (Stata Corporation, College Station, TX, USA).

## RESULTS

**FIG 2:**
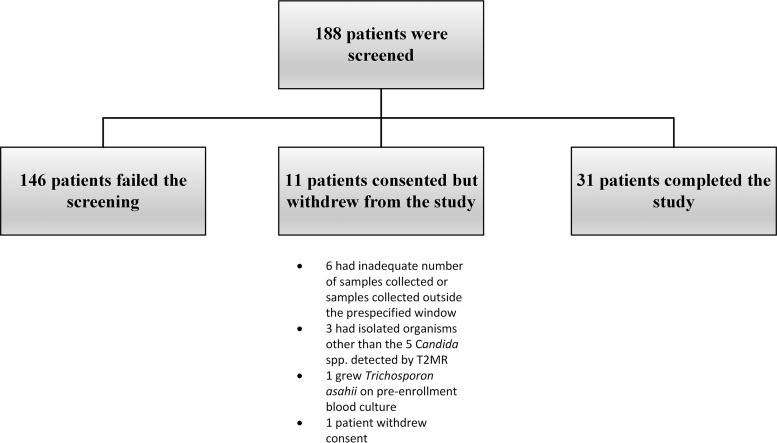
Flow chart of patients in the study.

Overall, 188 patients were screened, of whom 42 patients met the inclusion criteria and consented to participate in the clinical trial. Among those, 6 patients had either an inadequate number of samples collected or samples were collected outside the prespecified window, 3 patients had blood cultures with Candida spp. other than the 5 detected by the T2MR assay (1 Candida lusitaniae, 1 Candida guilliermondii, and 1 Candida dubliniensis), 1 withdrew from the study, and 1 eventually grew Trichosporon asahii, initially identified as yeast on the preenrollment blood culture, and was excluded from the study (Fig. 2). Thirty-one patients completed the study, and their data were used for the study analysis. All patients had a single Candida sp. isolated from their preenrollment blood culture, except for 1 patient with both C. albicans and C. parapsilosis. The frequencies of isolated Candida spp. among the 31 patients were C. glabrata, 12/31 (38.7%), C. albicans, 11/31 (35.5%), C. tropicalis, 4/31 (12.9%), C. parapsilosis, 2/31 (6.5%), C. krusei, 1/31 (3.2%), and C. albicans/C. parapsilosis, 1/31 (3.2%) (Table 1).

**TABLE 1:**
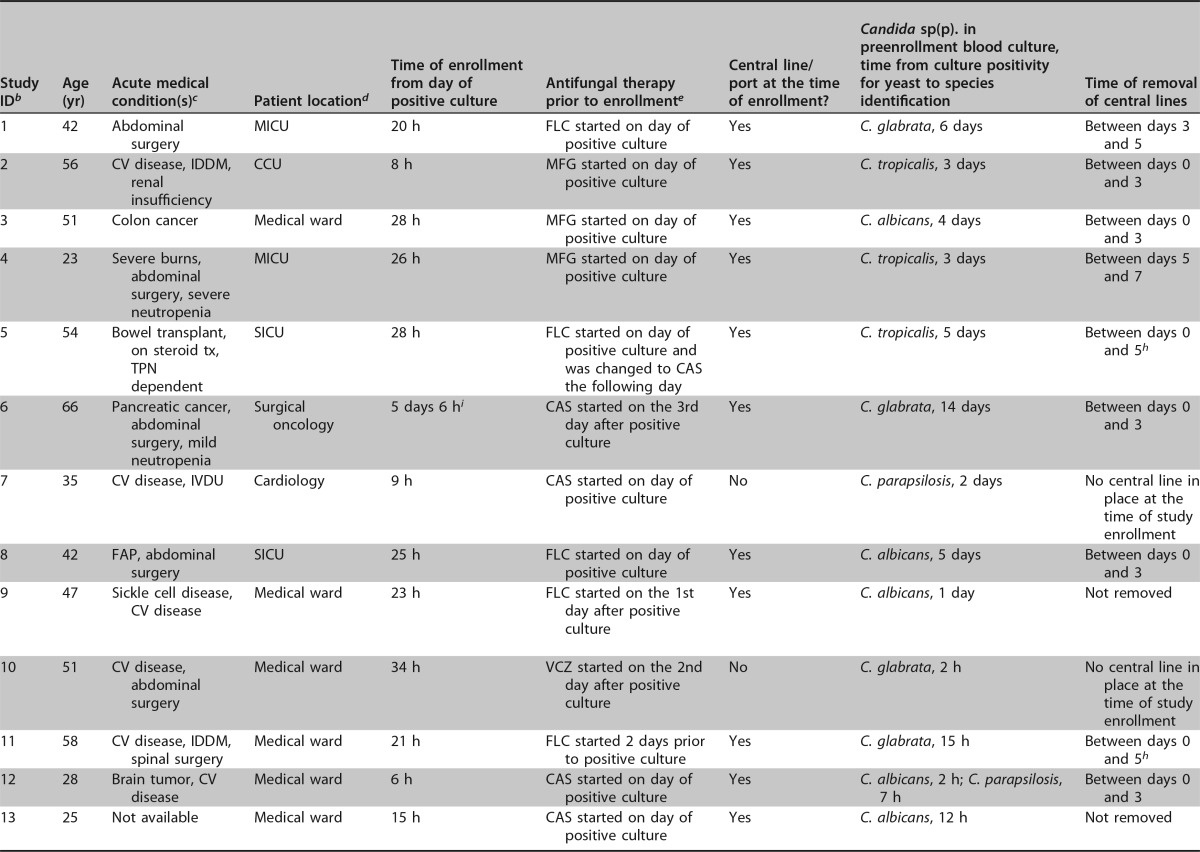

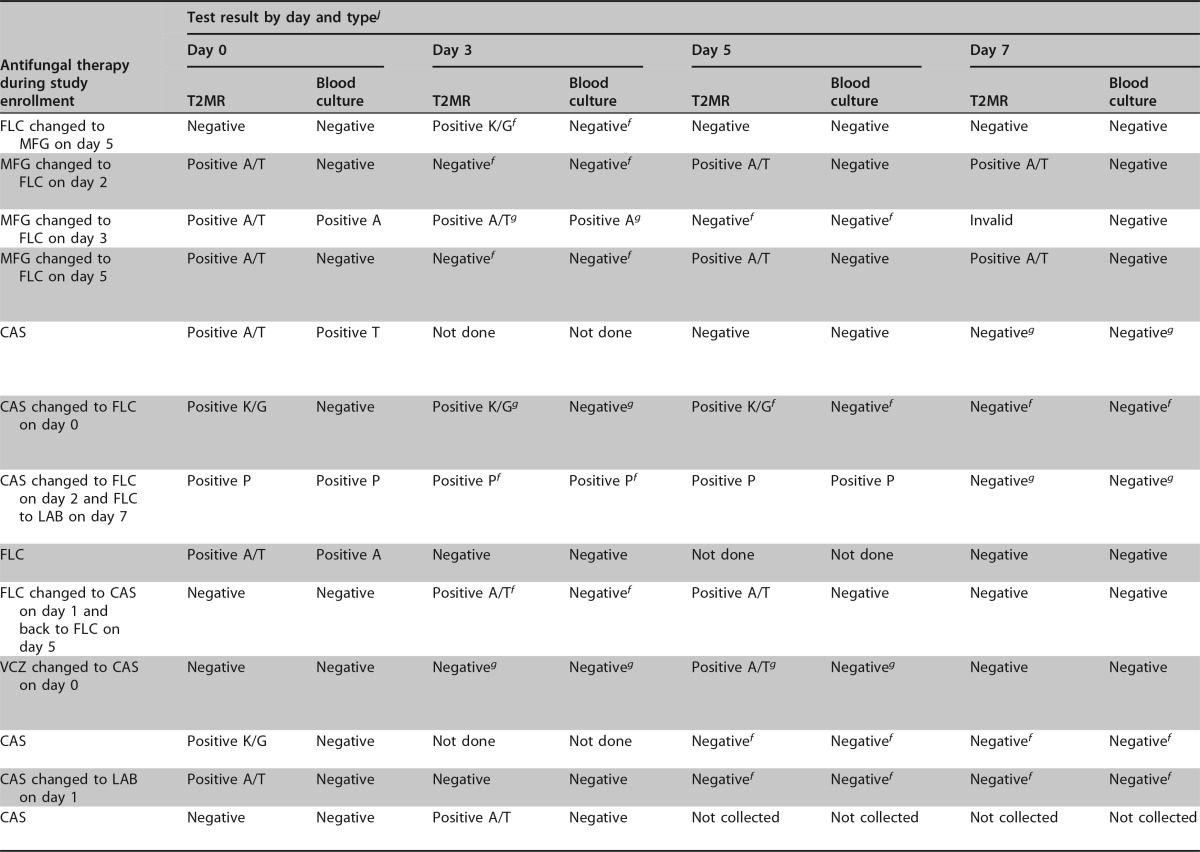
Baseline characteristics of study participants with positive surveillance T2MR and/or blood culture results and individual T2MR and blood culture results for subjects with at least 1 positive surveillance test result over the first week of monitoring^*a*^

a Days are defined from study enrollment.

^b^ ID, identification.

^c^ CV, cardiovascular; IDDM, insulin-dependent diabetes mellitus; tx, therapy; TPN, total parenteral nutrition; IVDU, intravenous drug use; FAP, familial adenomatous polyposis.

^d^ MICU, medical intensive care unit; CCU, coronary care unit; SICU, surgical intensive care unit.

^e^ FLC, fluconazole; MFG, micafungin; CAS, caspofungin; LAB, liposomal amphotericin B; VCZ, voriconazole.

^f^ Samples were collected within a +1-day window from the prespecified study visit to account for weekends and holidays.

^g^ Samples were collected within a −1-day window from the prespecified study visit to account for weekends and holidays.

^h^ Day 3 visit was not performed.

^i^ Protocol deviation regarding timing of study enrollment.

^j^ A, C. albicans; P, C. parapsilosis; T, C. tropicalis; A/T, C. albicans/C. tropicalis; K/G, C. krusei/C. glabrata.

In 18 patients (58.1%), all surveillance T2MR specimens and blood cultures collected for the purposes of the study were negative. Patient data for the remaining 13 patients (41.9%), who had at least one positive surveillance blood culture or T2MR test result, are outlined in Table 1. In total, out of the 93 sets of blood cultures and T2MR specimens that were collected, 7 blood cultures (7.5%) versus 23 T2MR specimens (24.7%) were positive (*P* = 0.001) in 4 (12.9%) and 13 (41.9%) unique patients, respectively (*P* = 0.01) (Table 1). Of note, all positive surveillance blood cultures had a positive accompanying T2MR result with concordance in the identified Candida sp. (7/7 [100%]), compared to only 7/23 (30.4%) positive T2MR results with an accompanying positive blood culture (Table 1). In 4 out of 13 patients, T2MR specimens and blood cultures were not collected on at least one of the prespecified study visits (on one visit in patients 5, 8, and 11 and on two visits in patient 13). This was due to visits falling on holiday weekends or due to device malfunction. In patient 3, the T2MR assay result was invalid on day 7 on both tubes A and B.

**FIG 3:**
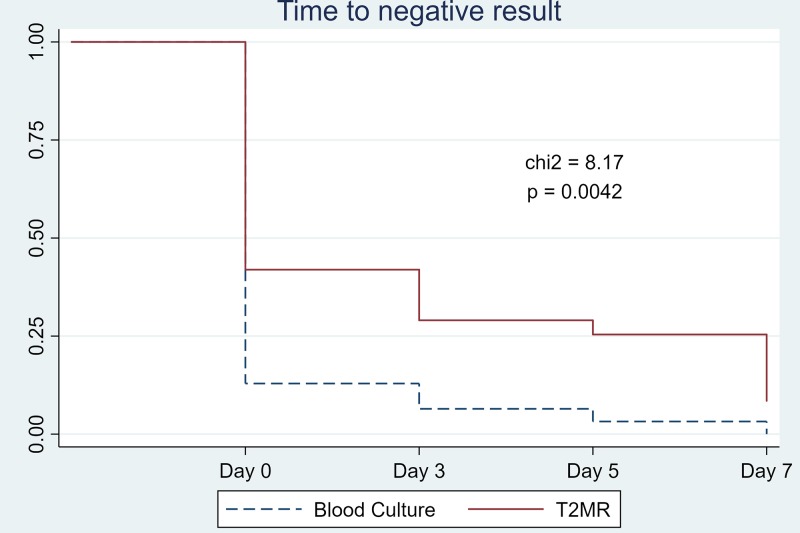
Kaplan-Meier diagram for the first week of surveillance. chi2, chi-square test.

By the end of the first surveillance week, candidemia was still detected in 18.2% of patients (2/11) by the T2MR assay (patients 2 and 4) versus 0% by the blood cultures. The Kaplan-Meier curves showing the length of time that the 31 patients remained candidemic by the 2 diagnostic methods are presented in Fig. 3. Based on the log rank hypothesis, which was used to compare the time-to-negative-result distributions for the 2 surveillance methods, there was a statistically significant improvement in posttreatment surveillance using the T2MR test compared to the regular blood cultures (chi-square, 8.2; *P* = 0.004) (Fig. 3).

Given the intermittent presence of Candida species cells in the blood of patients with invasive candidiasis, we reviewed the data of the patients with positive T2MR results to analyze the frequency of detection of patients with ongoing infection during each one of the sampling days (Table 1). Among the total of 13 patients with positive T2MR results during the study, 9 patients had a positive result on day 0 (Table 1). The addition of a second blood draw on day 3 allowed the detection of 3 more patients with ongoing infection who had a negative T2MR result on day 0 (patients 1, 9, and 13), increasing the detection rate by 33.3%. On day 5, another patient with active infection but negative prior specimens was identified (patient 10), but that patient had a different Candida sp. (C. albicans) detected from the preenrollment blood culture (C. glabrata), with evidence to suggest that this represented a new deep-seated infection, with C. albicans isolated from a biloma drainage culture. Sampling on day 7 did not identify any patients who did not already have positive results from previous blood draws.

## DISCUSSION

Even though clinical practice guidelines support the use of follow-up cultures in order to monitor the clearance of candidemia and guide therapy (6), blood cultures are a suboptimal tool to guide the treatment of candidemia. In the STAMP trial, we studied the performance of the T2MR assay as a monitoring tool for mycologic response to antifungal therapy in patients with candidemia. We followed 31 candidemic patients during their treatment with antifungal agents. All positive surveillance blood cultures had an accompanying positive T2MR result with concordance in the identified Candida sp., while only 7/23 (30.4%) T2MR results had an accompanying positive blood culture. Interestingly, we found a statistically significant improvement in posttreatment surveillance using the T2MR test compared to the regular blood cultures based on the Kaplan-Meier curves, with 18.2% of patients (2/11) remaining candidemic by the end of the first surveillance week based on the T2MR assay compared to none based on the blood cultures. All T2MR results were in agreement in terms of species-level identification both with the paired and the preenrollment blood cultures, except for one patient (patient 10) for whom the T2MR sample on day 5 was positive for a different Candida sp. from the preenrollment blood culture, with evidence to support deep-seated infection from this species.

This study provides evidence that the T2MR assay might outperform blood cultures in monitoring the clearance of Candida spp. in candidemic patients who are on antifungal treatment. This could be at least partially explained by the fact that T2MR results are not suppressed by antifungal agents. Indeed, published studies have demonstrated a decrease in the performance of blood cultures in detecting candidemia in the presence of therapeutic levels of antifungal agents (9). While the T2MR assay detects whole cells and not cell fragments or free DNA, *in vitro* studies suggested that the result is not inhibited by antifungal agents (8). This observation was also supported in the current clinical trial by the fact that none of the 31 patients who were on antifungal treatment had persistently invalid T2MR results that, as explained in Materials and Methods, would indicate the potential inhibition of the T2MR assay by the presence of therapeutic levels of antifungal agents.

Another factor that may have contributed to the significantly improved performance of the T2MR assay could be the long time to positivity for specific Candida spp., such as C. glabrata, that frequently exceeds the typical 5-day period during which cultures are processed (16, 17). This becomes particularly important, as in an expanding number of clinical centers, non-albicans Candida spp. represent >50% of clinical isolates (18). Indeed, in this trial, C. glabrata was detected in almost 39% of patients (12/31) and was the most frequent isolate among the enrolled patients. All subsequent surveillance blood cultures of those patients were negative, in comparison to 5 positive surveillance T2MR results in 3 different patients (patients 1, 6, and 11).

The 2016 IDSA guidelines recommend empirical treatment with an intravenous echinocandin and subsequent transitioning to oral fluconazole as soon as surveillance cultures are negative (for patients who are clinically stable and have a fluconazole-susceptible isolate). Based on studies using data from autopsy-proven cases of invasive candidiasis, we know that the sensitivity of blood cultures for diagnosing invasive candidiasis is roughly 50%, in a large part because of the intermittent nature of candidemia in deep-seated infections (7). In this study, a requirement of two consecutive negative T2MR results to document the clearance of Candida from the bloodstream increased the possibility of detecting ongoing fungemia by 33.3%. This seems to indicate that more than one T2MR result would be needed to demonstrate candidemia clearance. However, future clinical trials with a collection of surveillance samples for longer periods are needed to determine the required number of negative results that would be enough to justify a mycologic response to therapy.

The rapid turnaround time of the T2MR assay (mean time to negative result, 4.2 ± 0.9 h; mean time to species identification, 4.4 ± 1.0 h [14]) compared to the 5 days that blood cultures take to finalize can hasten the adjustment of antifungal therapy by 3 days, even when requiring 2 consecutively negative T2MR results within 48 h and 1 negative blood culture to document clearance. Echinocandins, the recommended first-line treatment for candidemia, are generally well tolerated (19, 20). Deescalation to another class of antifungals can reduce the risk of resistance development, since echinocandin resistance is almost always associated with previous exposure and prolonged treatment courses (21). The faster transition to oral therapy would also be expected to have implications in reducing hospital costs by reducing the length of stay, among other reasons (22).

Additionally, the rapid turnaround time of the T2MR assay could allow for timely adjustment of the treatment plan. Mortality in candidemia is closely linked to source control, with mortality rates approaching 100% in patients with septic shock without timely source control (4). Central venous catheters (CVCs) are not always the source of infection (6). Persistently positive T2MR results in patients with a CVC may, however, be the determining factor in a decision to remove a CVC. Further, consistently positive results in a patient after CVC removal may point to deep-seated infection and alert the clinician to the need for an additional diagnostic work-up to rule out visceral candidiasis. Indeed, in our study, we observed one patient with a positive T2MR result 5 days after enrollment, following 2 negative surveillance T2MR specimens, and this correlated with the diagnosis of Candida biloma (patient 10). Even though in this case the bile drainage culture was already finalized as being positive for C. albicans 2 days prior to the collection of the T2MR sample, this finding indicates that the T2MR assay can detect deep-seated candidiasis and potentially guide treatment decisions in cases where deep culture data are not available.

It should be noted that using the T2MR assay for monitoring candidemia is limited by the ability to recognize only 5 Candida species. This is particularly important in the era of increasing prevalence of other Candida spp., especially Candida auris, worldwide. However, based on worldwide registry data, over 92% of invasive disease is still caused by the 5 Candida spp. detected by the T2MR assay (23). Indeed, out of the 43 patients who consented to participate in the study, only 3 (7%) patients were excluded due to the isolation of Candida spp. other than the ones detected by the T2MR assay. Another potential shortcoming of the T2MR assay is that it may detect nonviable whole Candida cells after initiation of antifungal therapy. This potentially explains the intermittent positive T2MR results (i.e., patients 2 and 4). Furthermore, the intermittent release of Candida cells into the blood of patients with deep-seated candidiasis or the different Candida burden during the disease course would be another plausible explanation, but further studies are needed to justify this. Weaknesses of the study include the small sample size and the fact that we did not use the T2MR results to guide clinical decision-making, so as to correlate the results with patient outcomes. Thus, it is uncertain whether incorporating T2MR monitoring into clinical practice for the management of known candidemia leads to improved clinical outcomes, a decrease in the duration of intravenous antifungal treatment, side effects from antifungals, and the emergence of resistant Candida spp.; this is to be shown in future studies. Furthermore, despite some evidence in this study that T2MR might be able to detect at least some cases of deep-seated candidiasis, future studies are needed to determine the performance of the T2MR assay with invasive candidiasis without candidemia. The above-mentioned concerns should be addressed in well-designed clinical trials which will incorporate T2MR monitoring into critical management decisions among patients with proven and suspected candidemia.

The incorporation of the T2MR assay in daily practice is anticipated to pose financial challenges to the hospital budget (24, 25). Bilir et al. studied the economic impact of incorporating the T2MR assay in diagnostic protocols in certain high-risk patient populations with a 3% prevalence of disease, such as critical care admissions, solid organ transplantation, hematopoietic stem cell transplantation, and oncology patients (26). The diagnostic strategy that incorporated the T2MR assay was estimated to result in potential annual savings of $5,858,448 and a 47.6% reduction of cost in a 500-bed hospital, with the savings mostly driven by a reduction in mortality and days of hospital stay.

In conclusion, the finding of this clinical trial that almost one-third of patients had ongoing fungemia after the first negative blood culture indicates the need to revisit the way we currently determine the duration of therapy starting from the first negative blood culture. The STAMP trial provides strong evidence that among patients on antifungal therapy, the T2MR assay can be used to detect ongoing candidemia in a more timely fashion than the traditionally used blood cultures. The T2MR assay has the potential to be a monitoring tool for patients with invasive candidiasis and, given its quick turnaround, may provide actionable information to adjust treatment.
